# Chemical Profiling of Two Italian *Olea europaea* (L.) Varieties Subjected to UV-B Stress

**DOI:** 10.3390/plants11050680

**Published:** 2022-03-02

**Authors:** Chiara Piccini, Claudio Cantini, Giampiero Cai, Diana C. G. A. Pinto, Artur M. S. Silva, Marco Romi, Maria Celeste Dias

**Affiliations:** 1 Department of Life Sciences, University of Siena, Via Mattioli 4, 53100 Siena, Italy; cai@unisi.it (G.C.); marco.romi@unisi.it (M.R.); 2 Institute for BioEconomy, National Research Council of Italy, 58022 Follonica, Italy; claudio.cantini@ibe.cnr.it; 3 LAQV-REQUIMTE, Department of Chemistry, University of Aveiro, 3810-193 Aveiro, Portugal; diana@ua.pt (D.C.G.A.P.); artur.silva@ua.pt (A.M.S.S.); celeste.dias@uc.pt (M.C.D.); 4 Centre for Functional Ecology, Department of Life Sciences, University of Coimbra, Calçada Martim de Freitas, 3000-456 Coimbra, Portugal

**Keywords:** UV-B radiation, olive tree, metabolomic, phenolic profile, lipophilic profile

## Abstract

The depletion of the stratospheric ozone layer due to natural and/or anthropogenic causes decreases the amount of UV-B radiation filtered, and consequently increases the risk of potential damage to organisms. In the Mediterranean region, high UV-B indices are frequent. Even for species typical of this region, such as the olive tree, the progressive increase in UV-B radiation represents a threat. This work aimed to understand how high UV-B radiation modulates the phenolic and lipophilic profile of olive varieties, and identify metabolites that enhance olive stress tolerance. Two Italian olive varieties were subjected to chronic UV-B stress, and leaves were analyzed by gas and liquid chromatography. The results indicated that the most representative phenolic and lipophilic compounds of Giarraffa and Olivastra Seggianese were readjusted in response to UV-B stress. The Giarraffa variety seemed better suited to prolonged UV-B stress, possibly due to the higher availability of flavonoids that could help control oxidative damage, and the accumulation of hydroxycinnamic acid derivatives that could provide strong UV-B shield protection. In addition, this variety contained higher levels of fatty acids (e.g., palmitic, α-linolenic, and stearic acids), which can help to maintain membrane integrity and accumulate more sorbitol (which may serve as an osmoprotectant or act a free-radical scavenger), terpenes, and long-chain alkanes, providing higher protection against UV-B stress.

## 1. Introduction

The stratospheric ozone layer is deteriorating due to natural and anthropogenic origin factors that decrease the amount of UV-B radiation filtered and consequently increase the damage to living organisms [1]. Although measures have been implemented to reduce the amount of chemicals released into the atmosphere and damage the ozone layer, the intensity of ultraviolet B (UV-B) radiation reaching the Earth’s surface is estimated to increase until the mid-21st century [2]. Excessive exposure to UV-B radiation has diverse negative impacts that include a wide range of morphological, physiological, and reproductive aspects in plants and animals, as well as humans. In addition, it can alter biogeochemical cycles, and act synergistically with other environmental problems (such as global warming, ocean acidification, and pollution), thereby deeply impacting ecosystems [1]. Concerning plants, although UV-B radiation represents only a small fraction of the solar radiation that reaches the Earth’s surface, it induces a photobiological effect relevant to the anatomy, morphology, physiology, and biochemistry of plants [3,4]. Sunlight provides the energy needed for plant growth, but intense light radiation (particularly in the UV-B spectrum) can induce stress responses that potentially can lead to severe damage to DNA, proteins, membrane lipids, and other cellular components [5]. Although plants have developed several repair mechanisms over time, the damage caused by UV radiation is still considerable [6].

The Mediterranean region is frequently exposed to high irradiance accompanied by elevated UV-B indices, particularly during the spring and summer seasons [7]. The olive tree (*Olea europaea* L.) is a typical species of the Mediterranean region with high socioeconomic importance. This species is well adapted to the harsh climatic conditions of this region, and its tolerance to drought has been extensively studied [8]. However, there is a lack of comprehensive knowledge about the metabolic changes involved in the tolerance strategies of olive trees to UV-B stress. It remains unclear how UV-B radiation can modulate the metabolome and which metabolic changes enhance olive trees’ tolerance [9]. Functional changes that occur during stress are closely linked to metabolic network adjustments that, with the emergence of new metabolomic approaches, are beginning to be unraveled [10,11]. Indeed, the study of metabolomics has helped identify the most sensitive networks related to physiological adaptations in different species and find key stress metabolites [12]. In addition to studies on the model species Arabidopsis [13], a few crops have been studied for metabolome changes in response to UV-B, such as *Zea mays* [14] and *Lactuca sativa* [15]. In recent years, several studies have employed a metabolomic approach, allowing the identification of several groups of metabolites involved in the abiotic stress response (e.g., to UV-B, drought, and heat), such as epicuticular wax components (alkanes, terpenes, and fatty acids), membrane fatty acids, polyphenols, and terpenes [9,16,17,18,19,20,21]. However, much remains to be studied to fully understand the mechanisms of plant response to increased UV radiation and to determine its impact on other metabolic pathways [17].

Metabolomic studies in olive plants are scarce, and were mainly focused on the impact of abiotic stresses on central key pathways. These studies unraveled adjustments in important phenolic compounds (e.g., secoiridoids, flavonoids, and hydroxycinnamic acid derivatives), carbohydrates, and lipophilic metabolites related to cuticle wax (e.g., long-chain alkanes and terpenes), and identified some metabolites related to ROS scavenger action (e.g., thymol glycosides) and maintenance of membrane integrity (e.g., fatty acids and steroids) [9,16,17,22]. In previous works [23,24], we analyzed the physiological and biochemical responses of two economically important Italian olive varieties to UV-B stress. In the present study, the integration of metabolomics with these previous data allowed a deeper understanding of metabolite dynamics and their connection in a more extensive network of pathways involved in stress response. Thus, we hypothesized that UV-B radiation would promote changes in metabolic pathways, particularly in protective lipophilic and phenolic metabolites that may play an essential role in protection against UV-B stress. Therefore, gas chromatography–mass spectrometry (GC–MS) and ultrahigh-performance liquid chromatography–mass spectrometry (UHPLC–MS) analyses were undertaken in *O. europaea* leaves (Giarraffa and Olivastra Seggianese varieties) exposed to chronic UV-B stress (14 h per day for eight weeks).

## 2. Results

### 2.1. Phenolic Profile

The phenolic profile was evaluated in olive leaves (Giarraffa and Olivastra Seggianese varieties) of control plants and subjected to UV-B stress, sampled at the 2nd, 4th, 6th, and 8th week after the onset of stress. In the Giarraffa variety (Table 1), a total of 16 compounds were identified and quantified: 13 flavonoids, one secoiridoid, and two hydroxycinnamic acid derivatives. In the Olivastra Seggianese variety (Table 2), a total of 12 compounds were identified and quantified: 11 flavonoids and one secoiridoid.

ANOVA was able to underline a significant interaction between the factors of treatment and variety (Table S1) for apigenin 6,8-di-*C*-glucoside, apigenin, luteolin-7-*O*-rutinoside, luteolin-4′-methyl ether, luteolin-7-*O*-glucoside, luteolin, quercetin-3-*O*-glucoside, and diosmetin isomers 1 and 2. The stressed plants of both varieties present the highest (*p* ≤ 0.01) levels of these compounds (except for luteolin-7-*O*-rutinoside is. 1 and luteolin-7-*O*-glucoside not detected in stressed plants of the Giarraffa, and luteolin for Olivastra Seggianese). Moreover, the stressed plants of the Giarraffa variety show an increase in these compounds higher than the stressed plants of the Seggianese variety.

As regards dihydroquercetin, diosmetin isomer 3, and the two derivatives of hydroxycinnamic acids, these compounds were detected only in plants of the Giarraffa variety. An interaction between the factors of treatment and variety was also observed for these compounds, and they presented the highest (*p* ≤ 0.01) levels in stressed plants. Concerning caffeoyl-6′-secologanoside, it was detected only in the Olivastra Seggianese variety plants, and an interaction between the factors of treatment and variety was observed. The stressed plants presented the highest (*p* ≤ 0.01) levels of caffeoyl-6′-secologanoside.

Concerning the factors of treatment and sampling time, an interaction of these two factors was observed for all phenolic compounds identified (except for the oleuropein derivative, which was only present in control plants of Giarrafa; (Table S1). For apigenin 6,8-di-*C*-glucoside, luteolin-7-*O*-rutinoside (isomer 1 was not present in UV-B Giarrafa), luteolin-4′-methyl ether, luteolin-7-*O*-glucoside (not present in UV-B Giarrafa), luteolin, apigenin, and diosmetin isomers 1 and 2, the UV-B plants at T8 showed the highest levels. In Giarrafa, the UV-B plants at T8 also showed the highest levels of β-hydroxy-verbascoside, dihydroquercetin, quercetin-3-*O*-glucoside, and diosmetin isomer 3. For the Olivastra Seggianese, the highest levels of caffeoyl-6′-secologanoside and quercetin-3-*O*-rutinoside were found in UV-B plants at T8.

**Figure 1:**
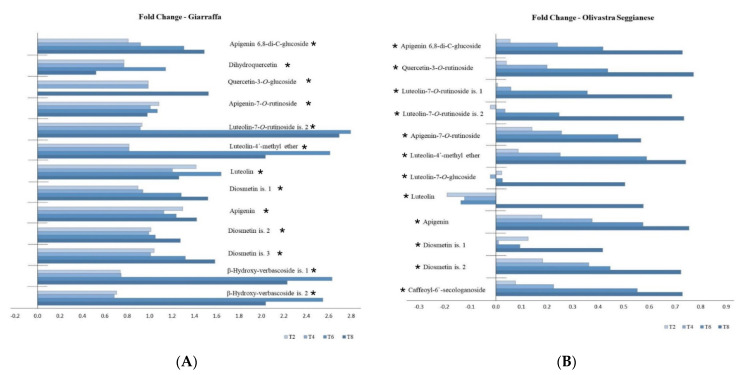
Fold changes (log_2_ (UV-B/control)) in phenolic metabolites of the Giarraffa (**A**) and Olivastra Seggianese (**B**) varieties after UV-B treatment sampled at the 2nd (T2), 4th (T4), 6th (T6), and 8th (T8) week after the onset of stress. ANOVA showed that most of chemical components were significantly affected by the main factors and their interactions. An asterisk (*) indicates a significant interaction between treatment vs. variety and treatment vs. sampling time.

Figure 1 shows the fold changes in phenolic metabolites of the Giarraffa (A) and Olivastra Seggianese (B) after UV-B treatment in the different sampling times (T2, T4, T6, and T8). In general, for the Olivastra Seggianese variety (Figure 1B), the profiles of the response of phenolic compounds progressively increased as stress progressed (except for the diosmetin isomer 1, luteolin, and luteolin 7-*O*-glucoside); while in the Giarraffa variety (Figure 1A), a more heterogenic profile of response was observed, with a progressive increase in some metabolites with the progress of stress, such as apigenin 6,8-di-*C*-glucoside, quercetin-3-*O*-glucoside, diosmetin isomers, and apigenin. In other phenolic metabolites, such as luteolin 7-*O*-rutinoside isomer 2, luteolin-4′-methyl ether, and β-hydroxyverbascoside isomers, the response was more intense in the T6 and T8.

### 2.2. Lipophilic Profile

The lipophilic profile was evaluated in olive leaves (Giarraffa and Olivastra Seggianese varieties) of control and UV-B stressed plants, sampled at the 2nd, 4th, 6th and 8th week after the onset of stress (Table 3 and Table 4). In the Giarraffa variety (Table 3), a total of 17 compounds were quantified: five terpenes, three carbohydrates, four fatty acids, and five alkanes. In the Olivastra Seggianese variety (Table 4), a total of 18 compounds were quantified: five terpenes, three carbohydrates, four fatty acids, five alkanes, and one sterol.

ANOVA showed (Table S2) a significant interaction between the factors of treatment and variety only for the lupeol derivative, with the plants of the Giarraffa variety under control and UV-B stress treatment presenting the highest levels of this terpene, followed by the Olivastra Seggianese UV-B-stressed plants. For the other lipophilic compounds (except the oleic acid derivative), an effect of the factor of treatment (control vs. UV-B stress) was observed, and the olive plants exposed to UV-B stress showed a level of these compounds significantly (*p* ≤ 0.05) higher than controls. In addition, an effect of the factor variety (Olivastra Seggianese vs. Giarraffa) also was observed for the compounds neophytadiene; palmitic and α-linolenic acids; long-chain alkanes 1, 2, 3, and 4; β-amyrin; and oleic acid derivative. Regarding the neophytadiene, Olivastra Seggianese had a significantly (*p* ≤ 0.05) higher compound content than Giarraffa. For the other compounds, Giarraffa had a significantly (*p* ≤ 0.05) higher content than Olivastra Seggianese.

Concerning the factors of treatment and sampling time, an interaction of these two factors was observed for all lipophilic compounds identified (Table S2). For the cases of neophytadiene, lupeol derivative, oleic acid derivative, and stigmast-5-en (only present in Giarraffa), the UV-B plants at T6 showed the highest levels, and for the other remaining compounds, they were more abundant in the UV-B plants at T8.

**Figure 2:**
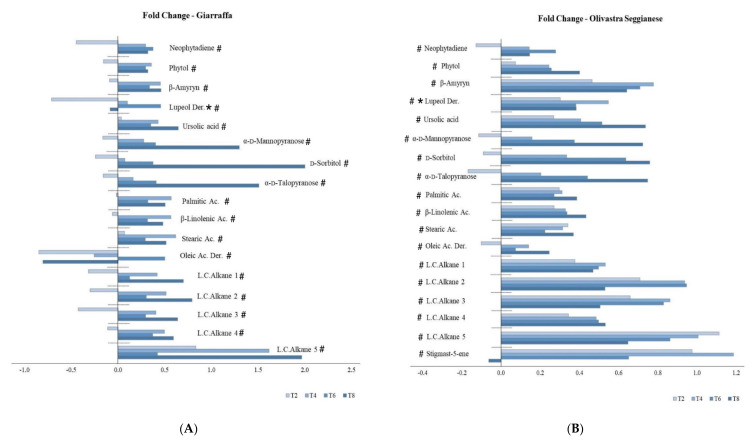
Fold changes (log_2_ (UV-B/control)) in lipophilic metabolites of the Giarraffa (**A**) and Olivastra Seggianese (**B**) varieties after UV-B treatment sampled at the 2nd (T2), 4th (T4), 6th (T6), and 8th (T8) week after the onset of stress. ANOVA showed that most of chemical components were significantly affected by the main factors and their interactions. An asterisk (*) indicates a significant interaction between treatment vs. variety; a hashtag (#) indicates a significant interaction between treatment vs. sampling time.

Figure 2 shows the rate of change in lipophilic metabolites of Giarraffa (A) and Olivastra Seggianese (B) after UV-B treatment at different sampling times (T2, T4, T6, and T8). In the Olivastra Seggianese variety (Figure 2B), the response profiles of some lipophilic metabolites progressively increased as stress progressed: phytol, ursolic acid, α-D-mannopyranose, D-sorbitol, α-D-thalopyranose, α-linolenic acid, and long-chain alkane 4. Other metabolites, however, did not show a progressive increase, but had peaks at T4 or T6 or both time points, as in the cases of neophytadiene; β-amyrin; lupeol derivative; long-chain alkanes 1, 2, and 3; and stigmast-5-ene. Others, such as palmitic and oleic acids, had peaks at T4 and T8. In addition, the stearic acid response intensity decreased progressively from T2 to T6 and then increased again slightly at T8, whereas the long-chain alkane 5 response intensity decreased with progressing stress from T2 to T8. In the Giarraffa variety (Figure 2A), the response profiles of some lipophilic metabolites increased progressively with stress: α-D-mannopyranose, α-D-thalopyranose, and D-sorbitol. Other metabolites, however, did not show a progressive increase, but had peaks at T4 and T8, such as phytol; β-amyrin; ursolic, palmitic, β-linolenic, and stearic acids; and long-chain alkanes 1, 2, 3, 4, and 5. For some lipophilic metabolites, such as f lupeol derivative and oleic acid derivative, a steady decrease, albeit with fluctuations, was observed in stressed samples compared to controls.

## 3. Discussion

### 3.1. Olive Plant UHPLC–MS Metabolite Profile

The metabolomic approach provided information on how the content of phenolic metabolites changed in response to UV-B stress, and identified specific compounds that appeared relevant to the olive response. Concerning the profile, some qualitative differences between the two varieties were detected. However, the flavonoids, secoiridoids, and hydroxycinnamic acids identified were already similar to those described for other olive varieties [25].

In the Olivastra Seggianese and Giarraffa varieties, flavonoids were the main phenolic compounds present in leaves. The flavonoid family is a vast group of compounds with different structures and roles [26]. Flavonoids are the principal phenols that contribute to the overall leaf antioxidant potency through ROS scavenging [27]. Some previous studies suggested that *o*-dihydroxy B-ring (catechol)-substituted flavonoids had a greater antioxidant capacity [28], and they could be found in several cell compartments near the centers of ROS generation or be transported from their sites of biosynthesis to these compartments [26,28]. Flavonoids can also prevent oxygen radical formation by inhibiting the activity of the enzymes involved in their generation [29]. In the Giarraffa variety, except for the luteolin-7-*O*-rutinoside isomer 1, luteolin-7-*O*-glucoside, and the oleuropein derivative, which were not detected in UV-B-stressed leaves, all the other flavonoids accumulated in response to the UV-B stress. These levels were, in general, higher than those found in Olivastra Seggianesse stressed plants. In turn, the Olivastra Seggianese variety showed a more heterogenic response to UV-B, but with a progressive increase in the majority of the flavonoids. The flavonoids, luteolin 7-*O*-rutinoside isomer 2, luteolin 7-*O*-glucoside, and luteolin in this variety, despite some fluctuations during the stress, achieved positive values at the end of the UV-B treatment (higher than those of the respective control). All these data suggested that both varieties responded to stress by increasing flavonoids pools, which represented a higher capacity to deal with the stress, particularly in the variety Giarraffa.

Some olive varieties are rich in luteolin-7-*O*-glucoside, a catechol B-ring-substituted flavonoid, which could be related to this species’ high tolerance to stress [16]. However, in others, the luteolin methylated forms (e.g., 4′-methoxyluteolin and 4′- or 3′-methoxyluteolin glucoside) seemed more responsive to stress, particularly the UV-B stress, decreasing their levels possibly due to their use in the neutralization of ROS [30], or due to the inactivation of the enzymes involved in the conversion of luteolin in their methylated forms [31]. In the present study, luteolin methylated forms were also found in both varieties, but their levels were consistently higher in stressed plants.

In general, the profiles of responses of flavonoids to UV-B obtained here for the two olive varieties were in line with those obtained in our previous studies, which also highlighted the accumulation of total flavonoids in response to UV-B radiation [23].

Another class of phenolic compounds identified as the secoiridoids. This family of compounds plays a crucial protective role in olive plants against drought, salt [32,33], and UV-B stress [17], suggesting some involvement in plant stress-defense mechanisms. Contrary to other studies with other olive varieties, only two secoiridoids were identified [17,34,35]. The decarboxymethyl oleuropein aglycone was only identified in the control plants of the Giarraffa variety. This compound is a derivative of oleuropein, which is one of the main phenolic compounds present in olive leaves [16]. Some studies reported the vital role of oleuropein in plant stress response, including to UV-B [17,25]. In turn, the other secoiridoid, caffeoyl-6-secologanoside, was only identified in Olivastra Seggianese, and UV-B plants showed a progressive increase in this metabolite compared to controls. In response to abiotic stresses, an accumulation of caffeoyl-6-secologanoside was also found in Cordovil of Castelo Branco olives [36], suggesting a possible protective stress role of this secoiridoid.

The last phenolic compound class identified was the hydroxycinnamic acid derivatives (HCAds). Hydroxycinnamic acids are predominantly involved in UV-B screening and accumulate mainly in the leaf epidermal cells, screening UV-B radiation that can reach photosynthetic tissues [27,37]. Moreover, they can also act as antioxidants through the scavenging of ROS, such as O_2_^•−^, OH^•^, and ^1^O_2_ [27]. In the present study, the HCAd β-hydroxyverbascoside was detected only in the leaves of the Giarraffa variety, and UV-B stress stimulated the production of this compound, which may provide extra UV-B shield protection [17]. These results suggested that the accumulation of β-hydroxyverbascoside in stressed plants allowed the Giarraffa variety to cope better with UV-B stress when compared with the Olivastra Seggianese. This was also in line with our previous work [23], in which Giarrafa showed a better performance (e.g., a higher photosynthetic efficiency) under prolonged UV-B stress. The β-hydroxyverbascoside was also identified in olive leaves from several other olive varieties [38].

### 3.2. Olive Plant GC–MS Metabolite Profile

The qualitative lipophilic profiles found in Olivastra Seggianese and Giarraffa were very similar. In Olivastra Seggianese leaves, the sterol stigmast-5-ene was also present in relatively high amounts, but was not in Giarraffa. Moreover, similar compounds were found in other varieties, and the fatty acids and long-chain alkanes were the most representative compounds found in olive leaves [9,16,35].

Regarding fatty acids, UV-B stress stimulated the production of palmitic, α-linolenic, and stearic acids in both varieties. Furthermore, stressed plants of Giarraffa produced more palmitic, α-linolenic, and stearic acids than the stressed plants of Olivastra Seggianese. This suggested that the UV-B stress response was weaker in Olivastra Seggianese, and it is possible this variety was unable to engage the same defense mechanisms as Giarraffa, based on readjustments of the levels of the fatty acids. These results aligned with our previous work, in which lipid peroxidation was only noticeable in the Olivastra Seggianese variety (particularly from T2 to T8) [24]. The absence of variations in lipid peroxidation [24] in stressed Giarraffa plants could be related to the higher palmitic, α-linolenic, and stearic acid contents found in these plants. Indeed, fatty acids have been described as constitutive elements of complex lipids, but several studies also suggested their direct involvement in abiotic and biotic responses to stress in plants [39,40]. Complex lipids, in turn, play an essential role in the structure and functions of cells by maintaining the integrity of cells and organelles [41].

Mannitol is one of the main polyols usually found in olive leaves [42]. In the present work, we identified sorbitol, an isomer of mannitol, and the UV-B stress induced by the accumulation of this polyol in both varieties. This response profile aligned with our previous work, particularly for the Giarrafa variety, in which mannitol levels increased after UV-B stress [23]. Olive trees are well adapted to regions with high irradiance (particularly UV). Maintaining high levels of polyols may be essential to cope with this stress, since these compounds provide more efficient use of carbon, act as osmolytes, and defend against photo-oxidative damage [23,42]. Besides mannitol, UV-B stress also induced an accumulation of the carbohydrates α-D-mannopyranose and α-D-talopyranose up to T4 in stressed plants of both varieties. Carbohydrates are involved in several stress-protective mechanisms, and their increase following stress is a typical response of olive trees, particularly under drought [35]. An accumulation in the sugar pool can increase carbon and energy availability to cope with stressful conditions or decrease sugar use for growth [43].

UV-B treatment also induced adjustments in the levels of triterpenes (neophytadiene, phytol, β-amyrin, lupeol derivative, and ursolic acid) and long-chain alkanes in both varieties. Considering that the main components of olive leaf cuticular wax are triterpenes (e.g., ursolic acid and α– and β–amyrin), long-chain alkanes, alcohols, aldehydes, and fatty acids [20,44,45], we hypothesized that olive plants invested (increased the levels) in these compounds to strengthen the cuticle structure. This improvement provided a protective barrier against UV radiation, increased light reflectance, decreased UV radiation penetration into the mesophyll, and reduced membrane damages [9,46,47]. In addition, morphoanatomical studies performed in olive leaves showed an increase in cuticle thickness in response to a long period of UV-B exposure [48]. Furthermore, Giarraffa stressed plants produced more β-amyrin, lupeol derivative, and long-chain alkanes (1–4) than Olivastra Seggianese stressed plants. In particular, long-chain alkanes in Giarraffa stressed plants showed values two times higher than those found in Olivastra Seggianese stressed plants. This suggested that the higher content of terpenes and long-chain alkanes may have allowed Giarraffa stressed plants to tolerate the UV-B radiation better than the Olivastra Seggianese stressed plants.

## 4. Materials and Methods

### 4.1. Plant Growth Conditions and Application of UV-B Treatment

Olive trees (*Olea europaea* L.) that were 18 months old (both Olivastra Seggianese and Giarraffa varieties) were taken from the nursery of the Società Pesciatina di Orticoltura (Pescia, PT, Italy). Subsequently, plants were transferred to climatic cells with the following environmental conditions: temperature of 21 °C; relative humidity (RH) of 60%; photoperiod of 14 light h, 10 dark h [49]; light intensity of 500 µmol m^−2^ s^−1^; watering with 400 mL of water for each plant once a week; and commercial substrate type “Vigor Plant Soil” (Vigorplant Italia Srl, Fombio, Italy) [23]. Ultraviolet radiation was provided by two TL20W/12 lamps (Philips, Milano, Italy) that emitted in the wavelength of UV-B rays, and that had already been widely used and described in the literature; lamps were used exactly according to the protocol of Allen et al. [49]. Plants (*n* = 16 for each variety) were positioned under UV-B lamps in the climatic cell. Every day, the homogeneity of UV-B radiation emitted by lamps was verified using a Power Meter 840 with an 818-UV sensor (Newport Optical, Newport Beach, CA, USA). The UV-B biologically effective dose (BED), 25 kJ m^−2^ d^−1^, was calculated according to Correia et al. [50]. Control plants (*n* = 16 for each variety), present in the same climatic cell, were carefully separated from those treated by means of a plasterboard panel that shielded most of the UV radiation (BED of 1 kJ m^−2^ d^−1^). The UV-B treatment corresponded to a high UV-B dose, but within the natural values already reported on the earth’s surface [51]. The UV-B treatment was carried out for a period of 8 weeks for 14 h a day. During the treatment, eight time points were established: the first one before the onset of UV-B treatment (T0); and after 1, 2, 3, 4, 5, 6, 7, and 8 weeks of UV-B treatment (respectively indicated as T1, T2, T3, T4, T5, T6, T7, and T8) [23]. Leaf samples were collected at four representative sampling times (T2, T4, T6 and T8), immediately frozen in liquid nitrogen, and stored at −80 °C.

### 4.2. Preparation of Leaf Extracts

Frozen olive leaves were macerated and mixed with *n*-hexane (5 g of leaves for 50 mL of extraction solvent) at room temperature with magnetic stirring for 48 h. The *n*-hexane was removed, and a new extraction cycle of 24 h was performed with the addition of new *n*-hexane in the same volume. The *n*-hexane from the two extraction cycles was placed in a glass balloon, and the *n*-hexane was evaporated to dryness at low pressure in a rotatory evaporator. The extracts obtained were left to dry for one week. The pellet obtained was also left to dry, and then mixed with 50 mL of methanol to extract phenolic compounds. After a first extraction cycle of 48 h at room temperature with magnetic stirring, the methanol was removed, and new methanol was added for a second extraction cycle. This last cycle lasted 24 h. The methanol obtained from both extraction cycles was put together in a glass balloon and evaporated to dryness at low pressure in a rotatory evaporator. The extract was left to dry for two weeks.

### 4.3. Gas Chromatography−Mass Spectrometry

The extracts obtained from the *n*-hexane extraction were weighted and prepared for silylation. In a glass tube, 200 μL of the extract was mixed with 200 μL of tetracosane 0.5 mg mL^−1^, 250 μL of pyridine, 250 μL of *N*,*O*-bis(trimethylsilyl)trifluoroacetamide, and 50 μL of trimethylsilyl chloride and incubated at 70 °C for 40 min. A sample (1 µL) of the silylated extract was injected into the gas chromatography−mass spectrometry (GC−MS) device (QP2010 Ultra Shimadzu). The chromatography conditions were set as described in Dias et al. (2019). For the identification of the lipophilic compounds, the peaks obtained in the chromatograms were compared with the library entries of the mass spectra database (NIST14 Mass Spectral Library and Wiley Registry^®^ of Mass Spectral Data) or compared with the mass spectra and retention times of pure compounds analyzed and prepared similarly to the samples. Calibration curves were prepared for quantification with standard compounds representing the main families presented in the extracts (maltose for carbohydrates, palmitic acid for fatty acids, octadecane for alkanes, and cholesterol for terpenes) and obtained by injection of known concentrations of these standard compounds. For maltose, the concentrations ranged from 0.06 to 11 mg/mL (y = 0.042x + 0.013 and r^2^ = 0.99); for palmitic acid, they ranged from 0.3 to 9 mg/mL (y = 0.095x + 0.447 and r^2^ = 0.99); for octadecane, they ranged from 0.08 to 8 mg/mL (y = 0.092x + 0.082 and r^2^ = 0.98); and for cholesterol, they ranged from 0.04 to 0.7 mg/mL (y = 0.060x + 0.048 and r^2^ = 0.99). The results obtained are expressed in g/Kg DW and presented as mean ± standard deviation of three independent analyses.

### 4.4. Ultrahigh-Performance Liquid Chromatography–Mass Spectrometry

The dry methanolic extract was weighted, and 50 mg were collected and dissolved in 1 mL of methanol. Samples with a concentration of 10 mg mL^−1^ were filtered through a 0.2 mL nylon membrane (Whatman, Medstone, UK) and injected in the ultrahigh-performance liquid chromatography–mass spectrometry (UHPLC–MS) device (Thermo Scientific Ultimate 3000RSLC Dionex, Waltham, MA, USA). The chromatography analysis was performed as described by Dias et al. [16]. The UHPLC–MS equipment contained a Dionex UltiMate 3000 RS diode array detector coupled to a mass spectrometer, and a Thermo Scientific Hypersil GOLD column (1000 mm × 2.1 mm) with a particle size of 1.9 µm and a temperature adjusted to 30 °C. The mobile phase was composed of degassed and filtered acetonitrile and 0.1% formic acid (*v*/*v*) at a flow rate of 0.2 mL min^−1^. During the first 14 min, the gradient of the solvent was 5% acetonitrile, followed by 40% formic acid for 2 min, 100% for 7 min, and 5% for the last 10 min. Then, 1 µL of the sample was injected into the UHPLC–MS device. UV–vis spectral data were collected between 250 and 500 nm wavelengths, and the chromatograms were recorded at 280 nm. The equipment contained a mass spectrometer (LTQ XL Linear Ion Trap 2D) with an orthogonal electrospray ion source (ESI) that operated in negative-ion mode with an electrospray ionization source of 5.00 kV (ESI capillarity temperature of 275 °C). It covered a mass range of 50.00 to 2000.00 *m*/*z*, and collision-induced dissociation MS/MS and MS^n^ experiments were performed for precursor ions. The retention times, UV–vis spectra, and spectral data were compared with those of standard compounds to identify the phenolic compounds. Semiquantification was performed by peak integration through the standard external method, using the closest standard compound. The detection and quantification limits (LOD and LOQ, respectively) were determined using calibration curves prepared with standard compounds (each family: quercetin and luteolin for flavonoids, and caffeic acid for secoiridoid and hydroxycinnamic acid derivatives). Calibration curves were obtained by injection of known concentrations (ranging from 5µg to 0.5 mg/mL) of these standard compounds: quercetin (y = 3 × 10^−7^x + 0.0951 and r^2^ = 0.99), luteolin (y = 2 × 10^−7^x + 0.0236 and r^2^ = 0.98), and caffeic acid (y = 9 × 10^−8^x + 0.0358 and r^2^ = 0.99). The results obtained are expressed in g/Kg DW and presented as the mean ± standard deviation of four independent analyses.

### 4.5. Statistical Analysis

Statistical analysis was performed using the Systat 11 statistical package (Systat Software Inc., Richmond, CA, USA). Data were checked for normality distribution by the Shapiro–Wilk test before a repeated-measures ANOVA analysis to test the significance of each of the three factors: treatment, variety, and time, as well as their interactions. When ANOVA presented *p* ≤ 0.01 or 0.05, a post hoc test was performed, and the content of each dependent variable in the control and treated plants was discussed in relation to the variety and date.

## 5. Conclusions

This study characterized the most representative phenolic and lipophilic compounds in Giarraffa and Olivastra Seggianese leaves. We demonstrated how the levels of some compounds were modulated by UV-B stress and that, in general, UV-B plants at the end of the experiment (T8) contained higher levels of phenolic and lipophilic compounds. The Giarraffa variety seemed better suited to prolonged UV-B stress, possibly due to the higher availability of flavonoids that neutralized ROS and radicals, and the accumulation of HCAds that provided extra UV-B shield protection. Besides phenolic compounds, this variety also stood out due to the high levels of fatty acids (e.g., palmitic, α-linolenic, and stearic acids), which could help to maintain membrane integrity; accumulation of sorbitol, which may have osmoprotective and antagonistic functions against free radicals and increases in some terpenes; and long-chain alkanes, which could provide better protection from UV-B radiation. The investment in the synthesis of these phenolic and lipophilic compounds to cope with the UV-B stress may occur at the expenses of other processes. Besides the lipophilic and phenolic metabolite profiles, the expression and/or activity of key enzymes involved in the biosynthesis of these metabolites (e.g., phenylalanine ammonia lyase, chalcone-synthase, and acetyl-CoA carboxylase) deserve further studies to understand their roles in *O. europaea* response to and tolerance of UV-B stress.

## Figures and Tables

**Table 1:** Phenolic profile (mg kg^−1^ DW) of *Olea europaea* leaves (Giarraffa variety) under control (C) and UV-B conditions sampled at the 2nd (T2), 4th (T4), 6th (T6), and 8th (T8) week after the onset of stress. Values are mean ± standard deviation (*n* = 3–4). Rt—retention time; Nd—not detected; is.—isomer. ANOVA showed that most of chemical components were significantly affected by the main factors and their interactions.

Rt (min)	Compound	[M-H]^−^ (*m*/*z*)	MS^2^ (*m*/*z*) Fragments	T2		T4		T6		T8	
				C	UV-B	C	UV-B	C	UV-B	C	UV-B
**Flavonoids**											
9.8	Apigenin 6,8-di-*C*-glucoside	593	353/383/503/575	169.9 ± 15.3	297.6 ± 31.6	174.3 ± 8.49	329.4 ± 10.3	179.6 ± 7.2	443.8 ± 15.0	175.9 ± 4.6	493.4 ± 9.8
11.9	Luteolin-7-*O*-rutinoside is. 1	593	285/447	175.1 ± 15.5	Nd	183.0 ± 9.65	Nd	57.3 ± 10.1	Nd	87.1 ± 11.6	Nd
12.2	Dihydroquercetin	303	125/177/285	181.2 ± 18.8	309.1 ± 31.5	196.5 ± 12.1	335.6 ± 10.8	201.9 ± 18.3	445.9 ± 15.6	351.0 ± 36.7	503.9 ± 23.7
12.5	Quercetin-3-*O*-glucoside	463	301	170.3 ± 15.0	337.7 ± 39.3	174.5 ± 8.41	346.2 ± 10.9	180.1 ± 7.3	Nd	180.5 ± 4.9	519.9 ± 48.8
12.9	Apigenin-7-*O*-rutinoside	577	269	185.4 ± 17.8	392.2 ± 43.3	189.2 ± 10.5	379.6 ± 11.3	225.7 ± 35.6	473.7 ± 27.1	269.6 ± 24.0	531.4 ± 61.5
13.1	Luteolin 7-*O*-rutinoside is. 2	593	285/447	179.6 ± 18.2	342.8 ± 36.6	184.4 ± 9.71	347.7 ± 10.9	64.9 ± 18.2	450.6 ± 17.0	81.0 ± 16.9	523.1 ± 50.1
13.5	Luteolin-4′-methyl ether	607	284/299	188.1 ± 18.2	331.1 ± 35.2	194.8 ± 11.5	343.2 ± 10.8	74.3 ± 24.3	453.6 ± 19.1	130.8 ± 19.2	535.0 ± 67.6
13.6	Luteolin-7-*O*-glucoside	447	289	173.8 ± 15.9	Nd	180.0 ± 9.13	Nd	51.8 ± 7.1	Nd	70.0 ± 6.6	Nd
15.9	Luteolin	285		211.6 ± 21.4	563.4 ± 66.2	227.0 ± 19.0	522.7 ± 14.6	165.4 ± 75.7	514.8 ± 46.5	253.1 ± 36.8	606.9 ± 171.6
16.7	Diosmetin is. 1	299	284	171.3 ± 15.5	318.4 ± 34.4	174.9 ± 8.52	335.2 ± 10.9	183.2 ± 7.2	445.6 ± 15.8	174.9 ± 4.7	501.1 ± 18.4
17.6	Apigenin	269	149	176.3 ± 16.4	432.8 ± 39.7	179.2 ± 9.15	391.3 ± 11.2	196.5 ± 14.7	463.3 ± 23.2	202.4 ± 6.9	541.9 ± 77.2
17.9	Diosmetin is. 2	299	284	182.7 ± 15.2	368.1 ± 38.6	187.7 ± 10.4	373.5 ± 11.0	224.7 ± 33.8	465.8 ± 24.6	237.9 ± 17.9	575.5 ± 124.9
20.1	Diosmetin is. 3	299	284	169.7 ± 15.2	349.1 ± 35.5	174.1 ± 8.38	350.0 ± 10.8	179.9 ± 7.2	449.0 ± 17.1	178.2 ± 4.8	533.8 ± 66.6
**Secoiridoid**											
11.3	Decarboxymethyl oleuropein aglycone	319	183	175.2 ± 16.0	Nd	181.6 ± 9.58	Nd	96.5 ± 40.1	Nd	52.8 ± 6.0	Nd
**Hydroxycinnamic Acid Derivatives**											
10.6	β-Hydroxyverbascoside is. 1	639	529/621	177.0 ± 16.5	295.3 ± 31.7	195.2 ± 11.8	327.4 ± 9.6	72.2 ± 5.5	447.0 ± 16.1	104.7 ± 7.3	490.6 ± 7.6
10.7	β-Hydroxyverbascoside is. 2	639	529/621	181.7 ± 17.4	296.5 ± 31.9	204.5 ± 13.9	328.7 ± 10.1	76.7 ± 9.6	448.3 ± 16.8	119.9 ± 10.4	492.0 ± 8.9

**Table 2:** Phenolic profile (mg kg^−1^ DW) of *Olea europaea* leaves (Olivastra Seggianese variety) under control (C) and UV-B conditions sampled at the 2nd (T2), 4th (T4), 6th (T6), and 8th (T8) week after the onset of stress. Values are mean ± standard deviation (*n* = 3–4). Rt—Retention time; is.—isomer. ANOVA showed that most of chemical components were significantly affected by the main factors and their interactions.

Rt (min)	Compound	[M-H]^−^ (*m*/*z*)	MS^2^ (*m*/*z*) Fragments	T2		T4		T6		T8	
				C	UV-B	C	UV-B	C	UV-B	C	UV-B
**Flavonoids**											
9.8	Apigenin 6,8-di-*C*-glucoside	593	353/383/503/575	217.0 ± 13.0	225.6 ± 7.6	207.5 ± 18.4	245.3 ± 3.6	203.1 ± 1.4	271.4 ± 15.4	215.5 ± 6.2	357.0 ± 1.2
11.6	Quercetin-3-*O*-rutinoside	609	301	216.1 ± 12.5	222.4 ± 7.0	213.0 ± 12.9	244.6 ± 3.3	199.2 ± 1.1	269.7 ± 14.8	210.9 ± 6.9	360.2 ± 1.1
11.9	Luteolin-7-*O*-rutinoside is. 1	593	285	224.1 ± 16.8	225.1 ± 8.3	238.4 ± 4.0	248.4 ± 4.4	217.8 ± 5.5	279.1 ± 16.5	236.3 ± 10.8	380.5 ± 4.3
12.1	Luteolin-7-*O*-rutinoside is. 2	593	285/447	229.6 ± 20.6	226.1 ± 7.9	251.4 ± 8.0	257.7 ± 5.2	246.7 ± 19.4	292.7 ± 19.7	277.0 ± 22.2	460.9 ± 19.5
12.8	Apigenin-7-*O*-rutinoside	577	269	229.3 ± 20.4	252.9 ± 13.7	248.7 ± 10.8	297.1 ± 10.8	256.0 ± 7.7	356.5 ± 35.1	288.6 ± 19.1	427.3 ± 13.3
13.1	Luteolin-4′-methyl ether	607	299/284	225.5 ± 17.8	239.5 ± 11.2	230.4 ± 6.1	274.3 ± 14.1	225.8 ± 3.0	339.6 ± 46.8	247.9 ± 8.5	414.6 ± 14.4
13.4	Luteolin-7-*O*-glucoside	447	285	246.8 ± 33.5	250.7 ± 15.5	282.4 ± 16.1	278.0 ± 4.6	315.6 ± 22.0	321.0 ± 27.9	353.3 ± 30.2	501.1 ± 29.2
15.8	Luteolin	285		342.0 ± 112.1	299.7 ± 29.5	489.8 ± 96.0	449.7 ± 22.7	543.1 ± 104.2	493.9 ± 77.3	471.4 ± 51.2	702.8 ± 47.9
17.5	Apigenin	269	225/149/201	228.0 ± 19.7	258.3 ± 15.7	234.8 ± 5.9	304.8 ± 14.9	228.5 ± 7.4	340.4 ± 31.2	243.5 ± 9.2	410.8 ± 9.2
17.8	Diosmetin is. 1	299	284	270.0 ± 52.5	294.7 ± 24.8	317.2 ± 33.6	319.3 ± 18.7	359.4 ± 34.9	383.5 ± 37.4	389.1 ± 28.5	519.9 ± 27.1
20.1	Diosmetin is. 2	299	284	221.4 ± 15.7	251.2 ± 14.6	226.9 ± 7.7	292.0 ± 13.8	224.9 ± 2.2	306.4 ± 24.6	241.7 ± 11.3	399.1 ± 12.5
**Secoiridoid**											
12.5	Caffeoyl-6′-secologanoside	551	507/341/389/281	220.3 ± 14.5	232.2 ± 8.6	223.0 ± 8.5	260.8 ± 5.6	215.1 ± 1.7	315.4 ± 24.7	234.4 ± 10.6	388.3 ± 5.7

**Table 3:** Lipophilic profile (g kg^−1^ DW) of *Olea europaea* leaves (Giarraffa variety) under control (C) and UV-B conditions sampled at the 2nd (T2), 4th (T4), 6th (T6), and 8th (T8) week after the onset of stress. Values are mean ± standard deviation (*n* = 3). Rt—Retention time. ANOVA showed that most of chemical components were significantly affected by the main factors and their interactions.

Rt (min)	Compound	T2		T4		T6		T8	
		C	UV-B	C	UV-B	C	UV-B	C	UV-B
**Terpenes**									
34.1	Neophytadiene	0.463 ± 0.013	0.340 ± 0.000	0.451 ± 0.004	0.554 ± 0.002	0.421 ± 0.006	0.546 ± 0.003	0.500 ± 0.021	0.623 ± 0.002
42.1	Phytol	0.361 ± 0.001	0.324 ± 0.000	0.358 ± 0.001	0.459 ± 0.001	0.323 ± 0.000	0.397 ± 0.000	0.396 ± 0.001	0.493 ± 0.001
67.9	β-Amyrin	0.633 ± 0.003	0.594 ± 0.003	0.630 ± 0.004	0.862 ± 0.005	0.570 ± 0.005	0.719 ± 0.007	0.697 ± 0.010	0.959 ± 0.012
71.7	Lupeol derivative	1.777 ± 0.004	1.084 ± 0.005	1.763 ± 0.024	1.888 ± 0.036	1.604 ± 0.034	2.202 ± 0.029	1.936 ± 0.033	1.826 ± 0.014
73.3	Ursolic acid	1.167 ± 0.009	1.194 ± 0.003	1.163 ± 0.010	1.564 ± 0.015	1.072 ± 0.049	1.366 ± 0.013	1.284 ± 0.018	2.005 ± 0.002
**Carbohydrates**									
35.4	α-D-Mannopyranose	0.103 ± 0.003	0.092 ± 0.000	0.101 ± 0.002	0.122 ± 0.003	0.090 ± 0.000	0.118 ± 0.000	0.113 ± 0.004	0.280 ± 0.008
36.3	D-Sorbitol	0.130 ± 0.000	0.109 ± 0.001	0.129 ± 0.001	0.135 ± 0.001	0.117 ± 0.000	0.152 ± 0.001	0.143 ± 0.000	0.572 ± 0.001
37.7	α-D-Talopyranose	0.111 ± 0.000	0.099 ± 0.001	0.110 ± 0.000	0.123 ± 0.001	0.100 ± 0.000	0.132 ± 0.000	0.122 ± 0.000	0.348 ± 0.003
**Fatty acids**									
39.2	Palmitic acid	3.017 ± 0.008	2.978 ± 0.006	2.992 ± 0.008	4.439 ± 0.008	2.731 ± 0.001	3.406 ± 0.003	3.347 ± 0.010	4.742 ± 0.008
43.0	β-Linolenic acid	3.284 ± 0.002	3.151 ± 0.005	3.252 ± 0.008	4.815 ± 0.014	2.946 ± 0.002	3.669 ± 0.008	3.618 ± 0.005	5.042 ± 0.023
43.7	Stearic acid	2.664 ± 0.002	2.792 ± 0.002	2.640 ± 0.001	4.046 ± 0.002	2.423 ± 0.001	2.968 ± 0.000	2.977 ± 0.002	4.255 ± 0.004
72.7	Oleic acid derivative	1.490 ± 0.213	0.829 ± 0.036	1.593 ± 0.226	1.335 ± 0.014	1.578 ± 0.042	2.232 ± 0.034	1.771 ± 0.248	1.014 ± 0.019
**Alkanes**									
57.9	Long-chain alkane 1	1.341 ± 0.003	1.075 ± 0.007	1.331 ± 0.003	1.780 ± 0.006	1.217 ± 0.008	1.326 ± 0.004	1.491 ± 0.012	2.418 ± 0.021
62.4	Long-chain alkane 2	1.888 ± 0.001	1.534 ± 0.014	1.879 ± 0.010	2.683 ± 0.018	1.717 ± 0.025	2.117 ± 0.009	2.106 ± 0.018	3.644 ± 0.040
67.6	Long-chain alkane 3	2.694 ± 0.007	2.001 ± 0.008	2.676 ± 0.013	3.540 ± 0.031	2.418 ± 0.028	2.965 ± 0.012	2.970 ± 0.032	4.623 ± 0.034
70.2	Long-chain alkane 4	0.741 ± 0.011	0.685 ± 0.006	0.750 ± 0.041	1.059 ± 0.006	0.692 ± 0.014	0.894 ± 0.005	0.838 ± 0.003	1.266 ± 0.009
72.8	Long-chain alkane 5	0.566 ± 0.004	1.008 ± 0.059	0.568 ± 0.004	1.742 ± 0.030	0.526 ± 0.021	0.704 ± 0.017	0.632 ± 0.006	2.468 ± 0.064

**Table 4:** Lipophilic profile (g kg^−1^ DW) of *Olea europaea* leaves (Olivastra Seggianese variety) under control (C) and UV-B conditions sampled at the 2nd (T2), 4th (T4), 6th (T6), and 8th (T8) week after the onset of stress. Values are mean ± standard deviation (*n* = 3). Rt—Retention time. ANOVA showed that most of chemical components were significantly affected by the main factors and their interactions.

Rt (min)	Compound	T2		T4		T6		T8	
		C	UV-B	C	UV-B	C	UV-B	C	UV-B
**Terpene**									
34.1	Neophytadiene	0.533 ± 0.002	0.487 ± 0.006	0.530 ± 0.003	0.586 ± 0.006	0.578 ± 0.008	0.700 ± 0.003	0.483 ± 0.008	0.534 ± 0.007
42.1	Phytol	0.344 ± 0.001	0.361 ± 0.001	0.333 ± 0.001	0.395 ± 0.000	0.355 ± 0.002	0.424 ± 0.002	0.336 ± 0.002	0.444 ± 0.001
67.9	β-Amyrin	0.357 ± 0.001	0.492 ± 0.001	0.363 ± 0.008	0.621 ± 0.006	0.385 ± 0.015	0.629 ± 0.004	0.353 ± 0.001	0.551 ± 0.005
71.7	Lupeol derivative	1.290 ± 0.012	1.591 ± 0.046	1.281 ± 0.007	1.873 ± 0.009	1.378 ± 0.021	1.794 ± 0.017	1.143 ± 0.003	1.490 ± 0.026
73.3	Ursolic acid	1.007 ± 0.010	1.297 ± 0.064	1.083 ± 0.006	0.586 ± 0.015	0.578 ± 0.0008	1.647 ± 0.028	0.975 ± 0.006	1.626 ± 0.036
**Carbohydrates**									
35.5	α-D-Mannopyranose	0.120 ± 0.001	0.111 ± 0.000	0.117 ± 0.004	0.131 ± 0.001	0.121 ± 0.000	0.157 ± 0.001	0.116 ± 0.000	0.191 ± 0.004
36.3	D-Sorbitol	0.127 ± 0.000	0.119 ± 0.000	0.126 ± 0.000	0.159 ± 0.001	0.135 ± 0.000	0.209 ± 0.002	0.124 ± 0.000	0.209 ± 0.004
37.7	α-D-Talopyranose	0.134 ± 0.001	0.119 ± 0.000	0.133 ± 0.000	0.153 ± 0.001	0.141 ± 0.001	0.192 ± 0.001	0.131 ± 0.001	0.219 ± 0.002
**Fatty acids**									
39.2	Palmitic acid	2.777 ± 0.010	3.411 ± 0.003	2.766 ± 0.005	3.433 ± 0.009	2.946 ± 0.004	3.551 ± 0.015	2.961 ± 0.004	3.868 ± 0.013
43.0	β-Linolenic acid	2.870 ± 0.014	3.463 ± 0.003	2.834 ± 0.006	3.556 ± 0.004	3.024 ± 0.005	3.819 ± 0.005	2.949 ± 0.005	3.980 ± 0.015
43.7	Stearic acid	2.530 ± 0.005	3.202 ± 0.001	2.529 ± 0.001	3.144 ± 0.001	2.695 ± 0.002	3.145 ± 0.002	2.782 ± 0.002	3.592 ± 0.003
72.6	Oleic acid derivative	1.253 ± 0.004	1.167 ± 0.009	1.252 ± 0.003	1.380 ± 0.247	1.059 ± 0.024	1.114 ± 0.041	0.883 ± 0.006	1.046 ± 0.031
**Alkanes**									
52.9	Long-chain alkane 1	0.478 ± 0.001	0.621 ± 0.001	0.471 ± 0.001	0.681 ± 0.002	0.503 ± 0.001	0.709 ± 0.001	0.492 ± 0.001	0.681 ± 0.001
57.5	Long-chain alkane 2	0.689 ± 0.005	1.125 ± 0.007	0.684 ± 0.002	1.310 ± 0.003	0.732 ± 0.002	1.409 ± 0.009	0.670 ± 0.001	0.967 ± 0.006
62.4	Long-chain alkane 3	1.034 ± 0.009	1.632 ± 0.007	1.041 ± 0.008	1.890 ± 0.008	1.117 ± 0.010	1.983 ± 0.019	0.992 ± 0.008	1.407 ± 0.020
70.2	Long-chain alkane 4	0.607 ± 0.001	0.770 ± 0.001	0.607 ± 0.003	0.850 ± 0.002	0.647 ± 0.002	0.913 ± 0.007	0.622 ± 0.001	0.899 ± 0.004
72.8	Long-chain alkane 5	0.530 ± 0.002	1.146 ± 0.058	0.531 ± 0.010	1.067 ± 0.0322	0.905 ± 0.049	1.645 ± 0.038	0.894 ± 0.008	1.402 ± 0.005
Sterol									
67.6	Stigmast-5-ene	1.192 ± 0.008	2.344 ± 0.039	1.198 ± 0.008	2.725 ± 0.004	2.030 ± 0.039	3.188 ± 0.014	1.761 ± 0.009	1.686 ± 0.038

## Data Availability

Data are available upon request due to restrictions.

## Supplementary Materials

The following are available online at https://www.mdpi.com/article/10.3390/plants11050680/s1, Table S1: ANOVA table produced with experimental data of UV treated olive plants belonging to two different varieties compared to the control and analysed in four different dates. When a chemical component was not detected in both varieties in every date only the variables treatment and date were used with a lost in degrees of freedom into the ANOVA and consequent missing data in the table., Table S2: ANOVA table produced with experimental data of UV treated olive plants belonging to two different varieties compared to the control and analysed in four different dates.
