# Supplementary material for: Chemical Profiling of Two Italian Olea europaea (L.) Varieties Subjected to UV-B Stress
Source: Plants (Basel). 2022 Mar 2;11(5):680. doi: 10.3390/plants11050680 (PMC8912780; doi:10.3390/plants11050680)
Supplement: Supplementary file 1 [file plants-11-00680-s001.zip › supplemental ANOVA table 1.pdf]

	Apigenin 6,8-di-C-glucoside	b-hydroxy-verbascoside isomer 1	b-hydroxy-verbascoside isomer 2	Luteolin 7-O-rutinoside isomero 1	Dihydroquercetin	Quercetin-3-O-glucoside	Quercetin-3-O-rutinoside	Luteolin 7-O-rutinoside isomero 2	Luteolin 4'-methyl ether	Luteolin-7-O-glucoside	Luteolin	Diosmetin isomer 1	Apigenin	Diosmetin isomer 2	Diosmetin isomer 3	Caffeoyl-6'-secologanoside	Apigenin-7-O-rutinoside
Control	192.8±2.63	137.3±4.47	145.7±4.77	177.4±11.5	232.6±6.48	176.4±7.25	209.8±2.60	189.3±13.9	189.7±5.30	209.2±19.6	337.9±15.2	255.0±5.42	211.16±5.33	218.5±7.45	175.5±8.22	223.2±3.45	255.6±5.51
Treated UVB	332.9±2.63	390.1±4.47	391.4±4.77	295.7±15.2	398.6±6.48	400.0±9.36	274.2±2.60	331.7±17.0	366.0±5.30	354.3±25.9	519.2±15.2	389.7±5.42	392.9±5.33	378.9±7.45	420.5±8.22	299.2±3.45	356.1±14.6
**TREATMENT**																	
p=	*0.000*	*0.000*	*0.000*	*0.000*	*0.000*	*0.000*	*0.000*	*0.000*	*0.000*	*0.000*	*0.000*	*0.000*	*0.000*	*0.000*	*0.000*	*0.000*	*0.000*
Seggianese	242.8±2.63								274.7±5.30		474.0±15.2	356.6±5.42	281.1	270.5±7.45			
Giarraffa	282.9±2.63								281.0±5.30		383.1±15.2	288.1±5.42	323.0	327±7.45			
**VARIETY**																	
p=	*0.000*								*0.410*		*0.000*	*0.000*	*0.000*	*0.000*			
Seggianese C	210.7±3.72								232.4±7.50		461.6±21.5	333.9±7.67	233.7±7.54	228.7±10.5			
Seggianese UVB	274.8±3.72								317.0±7.50		486.5±21.5	379.4±7.67	328.6±7.54	312.1±10.5			
Giarraffa C	174.9±3.72								147.0±7.50		214.3±21.5	176.1±7.67	188.6±7.54	208.2±10.5			
Giarraffa UVB	391.0±3.72								415.0±7.50		552.0±21.5	400.1±7.67	457.3±7.54	445.7±10.5			
**TREATMENT x VARIETY**																	
p=	*0.000*								*0.000*		*0.000*	*0.000*	*0.000*	*0.000*			
T2	227.5±3.72	*236.2*±6.31	*239.1*±6.75	*212.4*±19.8	*245.2*±9.16	*254.0*±10.3	219.2±3.68	*244.5*±19.6	*246.1*±7.50	*230.5*±33.9	*354.2*±21.5	*263.6*±7.67	*273.8*±7.54	*255.9*±10.5	*259.4*±11.6	*226.2*±4.88	*241.1*±7.79
T4	239.1±3.72	*261.3*±6.31	*266.6*±6.75	*254.4*±16.2	*266.1*±9.16	*260.4*±10.3	228.8±3.68	*237.8*±24.1	*260.7*±7.50	*254.6*±33.9	*422.3*±21.5	*286.6*±7.67	*277.6*±7.54	*270.1*±10.5	*262.0*±11.6	*241.9*±4.88	*318.1*±28.1
T6	274.5±3.72	*259.6*±6.31	*262.5*±6.75	*208.3*±19.9	*323.9*±9.16	*288.2*±5.92	224.5±3.68	*224.2*±24.1	*272.6*±7.50	*285.5*±27.7	*429.3*±21.5	*342.9*±7.67	*307.2*±7.54	*305.4*±10.5	*314.5*±11.6	*265.2*±4.88	*306.2*±7.79
T8	310.4±3.72	*297.7*±6.31	*306.0*±6.75	*271.1*±19.9	*427.4*±9.16	*350.2*±10.3	285.6±3.68	*335.5*±19.6	*332.1*±7.50	*356.4*±33.9	*508.6*±21.5	*396.3*±7.67	*349.7*±7.54	*363.5*±10.5	*356.0*±11.6	*311.3*±4.88	*357.9*±7.79
**TIME** *p=*	*0.000*	*0.000*	*0.000*	*0.000*	*0.000*	*0.000*	*0.000*	*0.002*	*0.000*	*0.000*	*0.000*	*0.000*	*0.000*	*0.000*	*0.000*	*0.000*	*0.000*
**TREATMENT x TIME** *p=*	*0.000*	*0.000*	*0.000*	*0.011*	*0.001*	*0.000*	*0.000*	*0.000*	*0.000*	*0.031*	*0.043*	*0.000*	*0.000*	*0.000*	*0.000*	*0.000*	*0.000*
**VARIETY x TIME** *p=*	*0.000*								*0.153*		*0.001*	*0.007*	*0.205*	*0.104*			

ANOVA table produced with experimental data of UV treated olive plants belonging to two different varieties compared to the control and analysed in four different dates. When a chemical component was not detected in both varieties in every date only the variables treatment and date were used with a lost in degrees of freedom into the ANOVA and consequent missing data in the table.
