# Supplementary material for: Chemical Profiling of Two Italian Olea europaea (L.) Varieties Subjected to UV-B Stress
Source: Plants (Basel). 2022 Mar 2;11(5):680. doi: 10.3390/plants11050680 (PMC8912780; doi:10.3390/plants11050680)
Supplement: Supplementary file 1 [file plants-11-00680-s001.zip › supplemental ANOVA table 2.pdf]

	Neophytadiene	AlphaDMannopyranose	DSorbitol	AlphaDTalopyranose	PalmiticAcid	Phytol	AlphaLinolenic Acid	StearicAcid	alkane1	alkane2	alkane3	BetaAmyrin	alkane4	Lupeolderivatives	Oleonicacidderivative	alkane5	UrsolicAcid
Control	0.495±0.010	0.110±0.002	0.129±0.001	0.123±0.003	2.942±0.039	0.351±0.004	3.097±0.052	2.655±0.034	0.916±0.091	1.296±0.127	1.868±0.174	0.499±0.029	0.688±0.016	1.522±0.056	1.360±0.063	0.644±0.032	1.122±0.018
Treated UVB	0.546±0.021	0.150±0.012	0.208±0.030	0.173±0.016	3.729±0.115	0.412±0.011	3.937±0.129	3.393±0.102	1.162±0.127	1.849±0.179	2.505±0.217	0.679±0.031	0.917±0.035	1.719±0.065	1.265±0.085	1.398±0.110	1.517±0.050
**TREATMENT**																	
p=	*0.019*	*0.002*	*0.010*	*0.003*	*0.000*	*0.000*	*0.000*	*0.000*	*0.003*	*0.000*	*0.000*	*0.000*	*0.000*	*0.007*	*0.312*	*0.000*	*0.000*
Seggianese	0.554±0.014	0.133±0.005	0.151±0.007	0.153±0.007	3.214±0.079	0.374±0.008	3.312±0.088	2.952±0.074	0.580±0.020	0.948±0.059	1.387±0.079	0.469±0.023	0.739±0.026	1.480±0.050	1.144±0.034	1.015±0.079	1.287±0.051
Giarraffa	0.487±0.017	0.127±0.012	0.186±0.031	0.143±0.016	3.457±0.144	0.389±0.012	3.722±0.153	3.096±0.132	1.498±0.083	2.196±0.132	2.986±0.156	0.708±0.027	0.866±0.040	1.760±0.063	1.480±0.089	1.027±0.138	1.352±0.060
**VARIETY**																	
p=	*0.003*	*0.635*	*0.243*	*0.550*	*0.046*	*0.204*	*0.003*	*0.135*	*0.000*	*0.000*	*0.000*	*0.000*	*0.001*	*0.000*	*0.001*	*0.920*	*0.225*
Seggianese C	0.531±0.010	0.119±0.001	0.128±0.001	0.135±0.001	2.863±0.027	0.342±0.003	2.919±0.022	2.634±0.033	0.486±0.004	0.694±0.007	1.046±0.014	0.364±0.004	0.621±0.005	1.273±0.026	1.112±0.046	0.715±0.056	1.072±0.019
Seggianese UVB	0.577±0.024	0.148±0.009	0.174±0.011	0.171±0.012	3.566±0.055	0.406±0.009	3.704±0.062	3.271±0.056	0.673±0.010	1.203±0.051	1.728±0.068	0.573±0.017	0.858±0.017	1.687±0.047	1.177±0.051	1.315±0.080	1.501±0.044
Giarraffa C	0.459±0.009	0.101±0.003	0.130±0.003	0.111±0.002	3.022±0.066	0.360±0.008	3.275±0.072	2.676±0.060	1.345±0.029	1.898±0.042	2.689±0.059	0.633±0.014	0.755±0.016	1.770±0.036	1.608±0.058	0.573±0.012	1.171±0.024
Giarraffa UVB	0.515±0.032	0.153±0.022	0.242±0.058	0.175±0.030	3.891±0.218	0.418±0.019	4.169±0.237	3.515±0.194	1.650±0.154	2.495±0.235	3.282±0.286	0.784±0.042	0.976±0.064	1.750±0.124	1.353±0.163	1.480±0.206	1.532±0.091
**TREATMENT x VARIETY**																	
p=	*0.802*	*0.356*	*0.267*	*0.381*	*0.484*	*0.815*	*0.672*	*0.346*	*0.457*	*0.721*	*0.768*	*0.721*	*0.806*	*0.008*	*0.092*	*0.185*	*0.524*
T2	0.456±0.002	0.107±0.001	0.121±0.001	0.116±0.001	3.046±0.002	0348±0.001	3.192±0.003	2.797±0.001	0.879±0.002	1.309±0.004	1.840±0.006	0.519±0.002	0.701±0.002	1.436±0.007	1.185±0.035	0.812±0.025	1.184±0.007
T4	0.530±0.002	0.118±0.001	0.137±0.001	0.129±0.001	3.408±0.002	0.386±0.001	3.614±0.003	3.030±0.001	1.066±0.002	1.639±0.004	2.287±0.006	0.619±0.002	0.816±0.002	1.701±0.007	1.390±0.035	0.977±0.025	1.311±0.007
T6	0.561±0.002	0.122±0.001	0.153±0.001	0.141±0.001	3.159±0.002	0.374±0.001	3.364±0.003	2.807±0.001	0.939±0.002	1.494±0.004	2.121±0.006	0.576±0.002	0.787±0.002	1.745±0.007	1.496±0.035	0.945±0.025	1.309±0.007
T8	0.535±0.002	0.175±0.001	0.262±0.001	0.205±0.001	3.729±0.002	0.417±0.001	3.897±0.003	3.401±0.001	1.271±0.002	1.847±0.004	2.498±0.006	0.640±0.002	0.906±0.002	1.599±0.007	1.179±0.035	1.349±0.025	1.472±0.007
**TIME**																	
p=	*0.000*	*0.000*	*0.000*	*0.000*	*0.000*	*0.000*	*0.000*	*0.000*	*0.000*	*0.000*	*0.000*	*0.000*	*0.000*	*0.000*	*0.000*	*0.000*	*0.000*
**TREATMENT x TIME**																	
p=	*0.000*	*0.000*	*0.000*	*0.000*	*0.000*	*0.000*	*0.000*	*0.000*	*0.000*	*0.000*	*0.000*	*0.000*	*0.000*	*0.000*	*0.000*	*0.000*	*0.000*
**VARIETY x TIME**																	
p=	*0.000*	*0.000*	*0.000*	*0.000*	*0.000*	*0.000*	*0.000*	*0.000*	*0.000*	*0.000*	*0.000*	*0.000*	*0.000*	*0.000*	*0.000*	*0.000*	*0.000*

ANOVA table produced with experimental data of UV treated olive plants belonging to two different varieties compared to the control and analysed in four different dates.
